# Barriers to Ferrate(VI) Application in Water and Wastewater Treatment

**DOI:** 10.1021/acs.est.3c09203

**Published:** 2024-02-08

**Authors:** Yang Deng, Hussein I. Abdel-Shafy

**Affiliations:** † Department of Earth and Environmental Studies, Montclair State University, Montclair, New Jersey 07043, United States; ‡ Water Research & Pollution Control Department, National Research Centre, El-Behous Street, Dokki, Cairo 12622, Egypt

**Keywords:** ferrate(VI), water treatment, wastewater treatment, chemical oxidation, barriers, technology adoption

The potential of ferrate(VI) (FeO_4_^2–^) in water and wastewater treatment has been explored since the 1970s.^1,2^ Its distinctive features, including multitreatment capabilities and minimal formation of disinfection byproducts (DBPs), set it apart from conventional treatment agents like chlorine and ozone.^3^ Over the past several decades, considerable academic resources have been invested in laboratory and pilot-scale investigations of ferrate(VI) to address wide-ranging water contaminants. However, its large-scale application remains quite limited. This gap between extensive research and real-world implementation underscores the need for identifying obstacles to ferrate(VI) application. Here we argue that five technical barriers prevent the water industry from establishing the necessary credibility for the full-scale application of ferrate(VI) in water and wastewater treatment.

*(1) Challenges of Ferrate(VI) Synthesis.* The bottleneck for ferrate(VI) application is the lack of practical synthesis devices. To be viable for large-scale treatment, ferrate(VI) generation should be reliable, cost-effective, energy-efficient, safe, environmentally compliant, and easy with minimal maintenance. However, the existing approaches face different technical and/or economic challenges. Off-site wet oxidation methods can readily produce ferrate(VI) in highly alkaline solutions, but the subsequent separation for high-purity ferrate(VI) powders is laborious and cost-prohibitive. Therefore, this approach, though suitable for laboratory use, is infeasible for real-world applications.

Alternatively, on-site syntheses of ferrate(VI) offer a practical solution, retaining the oxidation step to yield a concentrated ferrate(VI) solution, while eliminating the further separation and avoiding feedstock shipping and storage. However, wet oxidation-based on-site generation suffers from the presence of residual chlorine and substantial alkaline. Residual chlorine may lead to the formation of harmful chlorinated byproducts during the ferrate(VI) treatment. In another way, the on-site ferrate(VI) synthesis can be electrochemically achieved, but it encounters issues like electrode passivation, competitive oxygen evolution, demanding electrolyte and alkaline requirements, and significant energy usage.^4^ For example, electrochemical ferrate(VI) synthesis is typically more energy-intensive (approximately 0.5 to several kWh/mol) than ozone generation (<0.5 kWh/mol).

Furthermore, ferrate(VI) can be electrochemically produced *in situ* using high-oxygen overpotential anodes, such as boron-doped diamond electrodes, under circumneutral conditions, enabling simultaneous Fe(VI) synthesis and water treatment within a single electrochemical reactor. However, in addition to prohibitive electrodes, the *in situ* synthesis approach shares common challenges with other electrochemical water treatment processes, including a high level of energy consumption, competing side reactions on anodes, and the potential formation of toxic halogenated byproducts in the presence of halide anions. Moreover, the co-occurrence of other electrochemically driven treatment processes like direct electrochemical oxidation and the generation of other oxidants may complicate the assessment of ferrate(VI)’s specific role in water treatment.

*(2) Lack of Tailored Reactor Designs.* Ferrate(VI) has principally been investigated as an oxidant to degrade contaminants of emerging concern (CECs) in water. Therefore, it can be applied in a manner similar to other oxidants (e.g., potassium permanganate) in practice to enhance traditional water treatments. However, this approach inherently loses one of its most attractive advantages over others, the ability to perform various treatments within one step, due to the absence of reactor designs and operational methods specifically optimized for its multifunctionality.^5^

To harness the multipurpose agent, innovative reactors should be tailored to accommodate diverse treatment needs and hydraulic characteristics. Critical factors, including reaction kinetics and mechanisms, efficient mixing and mass transfer, flow rates and patterns, contact time, and the interplay between treatment processes, play pivotal roles in system development. Moreover, reactor design considerations must encompass other aspects, such as energy efficiency, operational safety, and environmental impacts, to ensure the overall success.

*(3) pH Adjustment.* The addition of ferrate(VI) tends to increase the pH and often requires additional pH adjustment, particularly during the treatment of water and wastewater that lack sufficiently high acidity. The pH adjustment can increase the treatment complexity and costs.

In laboratory-scale ferrate(VI) studies, the impacts of pH on water quality and treatment are often masked by the use of unrealistically high pH buffer chemicals. However, in real-world settings, ferrate(VI) dosing can increase the pH due to (1) FeO_4_^2–^ protonation (p*K* = 7.3), leading to water self-ionization and OH^–^ release, (2) production of OH^–^ from ferrate(VI) self-decomposition in water, and/or (3) the presence of excess alkaline in ferrate(VI) powders or concentrated solutions for stabilizing Fe(VI) during its synthesis. The resultant pH increase is collectively governed by different factors such as the initial pH, the acidity of water and wastewater, the ferrate(VI) dose, and the quantity of alkaline residuals along with ferrate(VI) chemicals. It is noteworthy that the first two aforementioned causes, that is, ferrate(VI) protonation and self-decomposition, are intrinsically linked to ferrate(VI) chemistry, with an estimated overall molar ratio of dosed ferrate(VI) to the yielded OH^–^ of 1:2 within the typical water and wastewater treatment-related pH range.^6^ For example, on the basis of carbonate equilibrium calculations, ferrate(VI) dosing from 0.5 to 10.0 mg/L Fe(VI) into water with an alkalinity of 200 mg/L as CaCO_3_ and an initial pH of 8.3 can result in an increase in the final pH from 8.5 to 9.4. When the water has a lower base-neutralizing capacity or substantial alkaline exists with the dosed ferrate(VI), a much larger increase in pH is anticipated.

Decreasing the pH may be necessary during or after treatment to meet regulatory compliance, influence pH-dependent chemical reactions, and modulate ferrate(VI) reactivity. Because wastewater treatment effluents typically tolerate a broader pH range than finished drinking water (e.g., pH 6.0–9.0 in the secondary wastewater treatment standards vs pH 6.5–8.5 in the national secondary drinking water regulations in the United States), adjusting the pH is more likely in drinking water treatment than in wastewater treatment. Overall, the additional pH adjustment, though common in practice, places ferrate(VI) treatment at a disadvantage against established water technologies.

*(4) Inadequate Information for Benchmarking against Established Treatment Technologies.* The water industry often hesitates to embrace new technologies unless their benefits are clear and compelling within specific application scenarios. However, the industry confronts significant limitations in obtaining data from side-by-side comparison experiments when assessing ferrate(VI) technology alongside existing treatment processes.

Unlike multifunctional ferrate(VI) treatment, most established treatment technologies concentrate on the removal of specific groups of contaminants. Consequently, existing comparative research tends to narrow the assessment to a specific treatment function, chemical oxidation in particular. Similar to ozone, ferrate(VI) exhibits selective chemical oxidation, albeit with limited mineralization of organic contaminants. It is known to preferentially attack the electron-rich moieties on CECs, such as phenolic, olefinic, aniline, and amine functional groups. However, ferrate(VI) degradation of CECs is more readily surpassed by coexisting NOM or other dissolved organic matter than ozonation.^7^ In the context of preoxidation, ferrate(VI) and ozone both demonstrate potential in mitigating the formation potentials of DBPs during the ensuing chlorination and chloramination, which is advantageous compared to permanganate.^8,9^ Nonetheless, they may lead to the formation of more assimilable organic carbon than other oxidants (e.g., chlorine dioxide, chlorine, and permanganate).^10^

The existing comparison studies cannot offer sufficient information for the industry to comprehensively gauge the technical viability, regulatory compliance, and economic advantages of ferrate(VI) technology. These assessments predominately rely on a specific treatment mechanism for the comparison, while overlooking co-occurring treatment functions during ferrate(VI) treatment. Moreover, the comparative studies were mostly conducted in controlled laboratory settings, but the information that the industry needs from larger-scale demonstration tests is lacking. This challenge can be attributed, in part, to the absence of large-scale ferrate(VI) generators. Finally, the currently available assessment studies primarily compare treatment effectiveness and efficiency between ferrate(VI) and other treatment processes. However, the data on other crucial factors that the industry weighs, such as energy consumption, costs, physical footprint requirements, system scalability, robustness and resilience of the treatment, ease of operation and maintenance, and regulatory compliance, have remained quite limited.

*(5) Management of Residuals.* A missing puzzle piece in ferrate(VI) studies pertains to the management of ferrate(VI) treatment residuals. Ferrate(VI) residuals primarily consist of iron (hydr)oxides with transport water and original impurities in water. The formation and separation of residuals from water contribute positively to iron removal to ensure compliance with water quality standards and prevent the emergence of reddish-brown water. However, the management of residuals can increase the system complexity and treatment expenses.

With established strategies for the management of residuals in conventional ferric coagulation treatment, the pressing question of whether ferrate(VI) residuals can be handled like ferric residuals, through either repurposing or disposal, arises. Unfortunately, our understanding of their formation, growth, and characteristics under water treatment conditions remains limited. In many laboratory-scale investigations, buffer chemicals are extensively used to maintain the pH and obscure solid formation. A limited number of studies show differences in the residuals produced by ferrate(VI) treatment compared to ferric addition.^11^ Moreover, conclusions about the characteristics of ferrate(VI) residuals, such as their amorphous and crystalline structural properties, are inconsistent due to variations in experimental conditions and analytical techniques.^11,12^ This knowledge gap not only affects solid–liquid separation but also challenges the management of residuals after ferrate(VI) application.

Identifying these barriers can guide future academic and industrial endeavors. The efforts are essential for overcoming these challenges, minimizing their impacts, and bridging the gap between research and the practical application of ferrate(VI) technology. Together, we can turn the promise of ferrate(VI) into a reality, revolutionizing the way water and wastewater are treated.

## References

[ref1] Gilbert M.; Waite T. D.; Hare C. An Investigation of the Applicability of Ferrate Ion for Disinfection. Journal of American Water Works Association 1976, 68 (9), 495–497. 10.1002/j.1551-8833.1976.tb02456.x.

[ref2] Waite T. D.; Gilbert M. Oxidative destruction of phenol and other organic water residuals by iron(VI) ferrate. J. - Water Pollut. Control Fed. 1978, 50 (3), 543–551.

[ref3] Sharma V. K.; Zboril R.; Varma R. S. Ferrates: Greener oxidants with multimodal action in water treatment technologies. Accounts of chemical research 2015, 48 (2), 182–191. 10.1021/ar5004219. 25668700

[ref4] Radjenovic J.; Sedlak D. L. Challenges and opportunities for electrochemical processes as next-generation technologies for the treatment of contaminated water. Environ. Sci. Technol. 2015, 49 (19), 11292–11302. 10.1021/acs.est.5b02414. 26370517

[ref5] Deng Y.; Guan X. Unlocking the potential of ferrate (VI) in water treatment: Toward one-step multifunctional solutions. Journal of Hazardous Materials 2024, 464, 132920 10.1016/j.jhazmat.2023.132920. 37988863

[ref6] Zheng L.; Cui J.; Deng Y. Emergency Water Treatment with Combined Ferrate (VI) and Ferric Salts for Disasters and Disease Outbreaks. Environmental Science: Water Research & Technology 2020, 6 (10), 2816–2831. 10.1039/D0EW00483A.

[ref7] Wang D.; Yu Y.; He J.; Ma J.; Zhang J.; Strathmann T. J. Comprehending the practical implementation of permanganate and ferrate for water remediation in complex water matrices. Journal of Hazardous Materials 2024, 462, 132659 10.1016/j.jhazmat.2023.132659. 37820527

[ref8] Jiang Y.; Goodwill J. E.; Tobiason J. E.; Reckhow D. A. Comparison of ferrate and ozone pre-oxidation on disinfection byproduct formation from chlorination and chloramination. Water Res. 2019, 156, 110–124. 10.1016/j.watres.2019.02.051. 30909124

[ref9] Rougé V.; von Gunten U.; Lafont de Sentenac M.; Massi M.; Wright P. J.; Croué J.-P.; Allard S. Comparison of the impact of ozone, chlorine dioxide, ferrate and permanganate pre-oxidation on organic disinfection byproduct formation during post-chlorination. Environ. Sci.: Water Res. Technol. 2020, 6 (9), 2382–2395. 10.1039/D0EW00411A.

[ref10] Ramseier M. K.; Peter A.; Traber J.; von Gunten U. Formation of assimilable organic carbon during oxidation of natural waters with ozone, chlorine dioxide, chlorine, permanganate, and ferrate. Water Res. 2011, 45 (5), 2002–2010. 10.1016/j.watres.2010.12.002. 21220144

[ref11] Goodwill J. E.; Jiang Y.; Reckhow D. A.; Gikonyo J.; Tobiason J. E. Characterization of Particles from Ferrate Preoxidation. Environ. Sci. Technol. 2015, 49 (8), 4955–4962. 10.1021/acs.est.5b00225. 25803182

[ref12] Lv D.; Zheng L.; Zhang H.; Deng Y. Coagulation of colloidal particles with ferrate (VI). Environmental Science: Water Research & Technology 2018, 4 (5), 701–710. 10.1039/C8EW00048D.

